# Mineral carbon pump in the Earth system

**DOI:** 10.1016/j.xinn.2024.100737

**Published:** 2024-11-19

**Authors:** Ke-Qing Xiao, Mingyu Zhao, Oliver Moore, Yao Zhao, Xin-Nan Li, Clare Woulds, Peyman Babakhani, Benjamin J.W. Mills, William B. Homoky, Karen Johnson, Alessandro Tagliabue, Chao Liang, Yong-Guan Zhu, Caroline Peacock

**Affiliations:** 1 State Key Lab of Urban and Regional Ecology, Research Center for Eco-Environmental Sciences, Chinese Academy of Sciences, Beijing 100085, China; 2 University of Chinese Academy of Sciences, Beijing 100049, China; 3 School of Earth and Environment, University of Leeds, Leeds LS2 9JT, UK; 4 State Key Laboratory of Lithospheric and Environmental Coevolution, Institute of Geology and Geophysics, Chinese Academy of Sciences, Beijing 100029, China; 5 Department of Environment and Geography, University of York, York YO10 5GY, UK; 6 State Key Laboratory of Environmental Criteria and Risk Assessment, Chinese Research Academy of Environmental Sciences, Beijing 100012, China; 7 School of Geography, University of Leeds, Leeds LS2 9JT, UK; 8 Department of Civil Engineering and Management, University of Manchester; Manchester M13 9PL, UK; 9 School of Engineering and Computing Sciences, Durham University, Durham DH1 3LE, UK; 10 School of Environmental Sciences, University of Liverpool, Liverpool L69 3BX, UK; 11 Institute of Applied Ecology, Chinese Academy of Sciences, Shenyang 110016, China

## Main text

**Figure 1:**
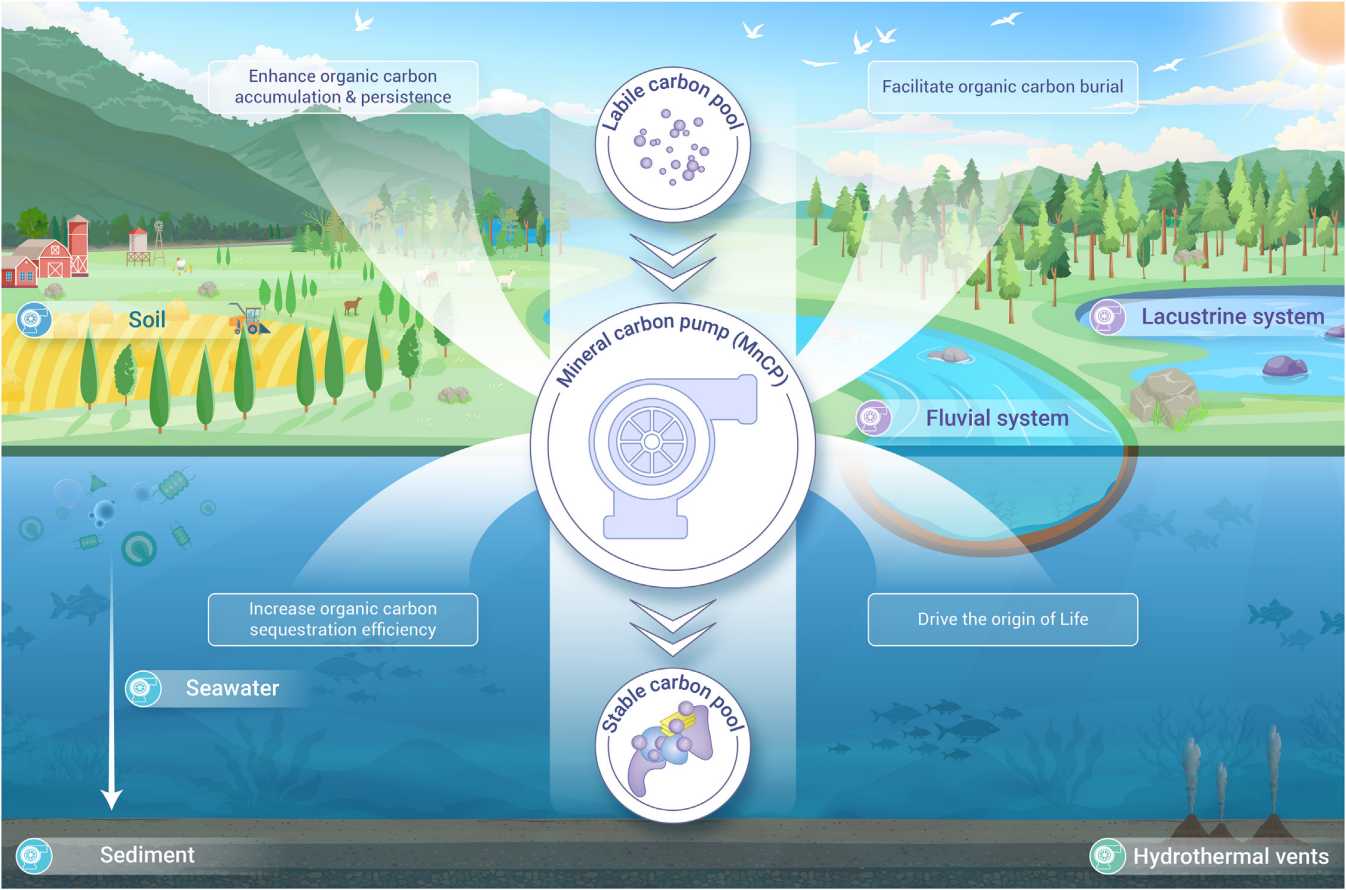
Mineral-OC interactions (MnCP) exist in multiple environments of the Earth system

The balance between the degradation and preservation of organic carbon (OC) is vital for the modulation of atmospheric CO_2_ and O_2_ in the Earth system, which regulates short-term climate as well as oxygenation of the early Earth. The mineral carbon pump (MnCP) was recently proposed to describe how soil minerals enhance the persistence and accumulation of OC, where interactions with minerals stabilize labile OC against microbial degradation (including via sorption, occlusion, aggregation, geopolymerization, and redox reactions).^1^ Given the widespread occurrence of metal (oxyhydr)oxides and clay minerals in terrestrial and marine environments and building on recent progress in mineral-OC interactions, we suggest that the MnCP occurs across the Earth system, where it plays a key role in OC preservation and hence the global carbon and oxygen cycles (Figure 1).

Contact between minerals and OC is inevitable in many terrestrial and marine environments, and interfacial reactions can happen spontaneously and commonly between minerals with charged hydroxyl groups, and/or permanent charge, and functional groups of OC (notably carboxyl, phenol, and amine). Metal (oxyhydr)oxides, particularly iron (oxyhydr)oxides, are typically an order of magnitude less abundant than clay minerals but are often disproportionately important for OC preservation because they have higher surface area and extremely reactive surfaces that facilitate dynamic interfacial reactions. Up to 80% of all OC across global terrestrial and marine depositional environments is preserved in association with reactive iron. In marine sediments, recent work finds that reactive iron and manganese minerals can geopolymerize labile OC molecules into more complex and recalcitrant macromolecular OC forms under ambient marine sediment conditions to bury around 4.1 Mt C year^−1^.^2^ Recent work also shows that in the ocean interior, the key role of the coupling between colloidal iron minerals and OC molecules is controlling iron distributions in seawater.^3^ This colloidal shunt mechanism highlights an important link between iron minerals and OC, like their coupling in marine sediments above. Thus, although oceanic primary production (PP) is often limited by iron, the MnCP can decouple PP from OC burial by enhancing preservation without requiring a concomitant increase in PP.

Clay minerals possess both charged hydroxyl groups and permanent surface charge and thus can also provide an important repository for OC in terrestrial and marine environments, where they may also occlude OC within their interlayers. Working together in concert, iron (oxyhydr)oxides/clay minerals can facilitate terrestrial OC burial during erosion and fluvial transit, which transfers biospheric particulate OC (POC) to downstream depositional basins or lakes, where mineral protection significantly reduces POC loss due to oxidation. Fluvial OC can also endure in marine environments when tightly associated with mica and chlorite,^4^ where it escapes disassociation that would otherwise occur when exposed to the high ionic strength of seawater. Once in the ocean, iron (oxyhydr)oxides/clay minerals can aggregate with algae to increase the carbon sequestration of the biological carbon pump, as mineral-OC aggregates usually sink faster in seawater and are thus less prone to microbial degradation. These findings strongly support the operation of the MnCP in environments other than soils, including fluvial and marine environments, where in the marine environment, the MnCP occurs in addition to the marine microbial carbon pump.

Such is the importance of the interactions between minerals and OC in the Earth system that the MnCP could have helped drive the origin of life and facilitated Earth’s biogeochemical evolution. Seafloor hydrothermal sediments and chimneys can provide flowthrough gradient systems (such as pH, temperature, and redox) that combine reactive minerals with organic compounds, which offer favorable conditions for redox and geopolymerization reactions that may have been instrumental in life’s beginnings. Reactive minerals like iron (oxyhydr)oxide or saponite clay can catalyze the synthesis of macromolecules (such as RNA and peptides) from smaller OC molecules in these environments. Given that the protection of OC via the MnCP results in the partial burial of OC, this allows O_2_ to accumulate in Earth’s early atmosphere. Through a compilation of the contents of iron oxides in fine-grained marine sediments throughout Earth history, recent works finds that the mean content of iron oxides of post-Tonian age (<830 Ma) is more than twice that of the pre-Cryogenian (>830 Ma)—0.43 ± 0.12 versus 0.19 ± 0.06 wt %.^5^ Along with a biogeochemical model, this work shows that an increase in the burial of OC associated with iron oxides could be a driver for the Neoproterozoic Oxygenation Event (NOE) and Great Oxidation Event (GOE). The increase in atmospheric oxygen could further promote the oxidation of pyrite in marine sediments to iron oxides, further accelerating OC burial and atmospheric oxygenation, thus providing a positive feedback on oxygenation.^5^

In summary, we posit that the MnCP is ubiquitous across many different Earth systems and should be included in multiple levels of modeling processes. Based on the variety of reactive minerals, including metal (oxyhydr)oxides and clays, the different types/sources of OC, and the array of environmental conditions present across the Earth system, we expect that different mineral-OC interactions (MnCP) likely dominate in different systems, leading to varied rates/efficiencies of the MnCP, which should now be quantitatively evaluated to assess their impact on OC preservation and burial, carbon and oxygen cycling, and related Earth system processes. Harnessing the power of the MnCP could also play a critical role in increasing OC stocks for climate change mitigation and achieving carbon neutrality.

## Acknowledgments

K.-Q.X. is funded by the CAS Youth Interdisciplinary Team and Hundred Talents Program of the Chinese Academy of Sciences. M.Z. is funded by the Hundred Talents Program of the 10.13039/501100002367Chinese Academy of Sciences C.L. is funded by the National Natural Science Foundation of China (32241037). This research project has also received funding from the 10.13039/501100000781European Research Council (ERC) under the European Union’s Horizon 2020 research and innovation programme (grant agreement no. 725613 MinOrg), and 10.13039/501100000270NERC Highlight Topic Grant (NE/S004963/1 Locked Up). C.P. acknowledges a 10.13039/501100000288Royal Society Wolfson Research Merit Award (WRM/FT/170005).

## Declaration of interests

The authors declare no competing interests.
